# *Mn-XRN1* Has an Inhibitory Effect on Ovarian Reproduction in *Macrobrachium nipponense*

**DOI:** 10.3390/genes14071454

**Published:** 2023-07-16

**Authors:** Tianyong Chen, Huwei Yuan, Hui Qiao, Sufei Jiang, Wenyi Zhang, Yiwei Xiong, Hongtuo Fu, Shubo Jin

**Affiliations:** 1 National Demonstration Center for Experimental Fisheries Science Education, Shanghai Ocean University, Shanghai 201306, China; chenty0523@126.com; 2 Wuxi Fisheries College, Nanjing Agricultural University, Wuxi 214081, China; yuan08102021@126.com; 3 Key Laboratory of Freshwater Fisheries and Germplasm Resources Utilization, Ministry of Agriculture, Freshwater Fisheries Research Center, Chinese Academy of Fishery Sciences, Wuxi 214081, China; qiaoh@ffrc.cn (H.Q.); jiangsf@ffrc.cn (S.J.); zhangwy@ffrc.cn (W.Z.); xiongyw@ffrc.cn (Y.X.)

**Keywords:** *Macrobrachium nipponense*, *XRN1*, ovarian reproduction, *VASA*, metamorphosis

## Abstract

*XRN1* is an exoribonuclease that degrades mRNA in the cytoplasm along the 5′–3′ direction. A previous study indicated that it may be involved in the reproduction of *Macrobrachium nipponense*. Quantitative real-time PCR was used to detect the spatiotemporal expression pattern of *Mn-XRN1*. At the tissue level, *Mn-XRN1* was significantly expressed in the ovary. During development, *Mn-XRN1* was significantly expressed at the CS stage of the embryo, on the 10th day post-larval and in the O2 stage of ovarian reproduction. The in situ hybridization results showed the location of *Mn-XRN1* in the ovary. The expression of *Mn-VASA* was significantly increased after in vivo injection of *Mn-XRN1* dsRNA. This suggests that *Mn-XRN1* negatively regulates the expression of *Mn-VASA*. Furthermore, we counted the number of *M. nipponense* at various stages of ovarian reproduction on different days after RNAi. The results showed that ovarian development was significantly accelerated. In general, the results of the present study indicate that *Mn-XRN1* has an inhibitory effect on the ovarian maturation of *M. nipponense*. The inhibitory effect might be through negative regulation of *Mn-VASA*.

## 1. Introduction

*M. nipponense* is an economically important freshwater aquaculture species in China due to the advantages of good stress resistance and rapid growth [1]. However, the rapid sexual maturity of *M. nipponense* has been a constraint on increased production. Female prawns have a short maturation cycle and can generally lay eggs two to three times per year [2]. Faster sexual maturity allows females to spend most of their energy on ovarian development, resulting in smaller individuals [3]. Furthermore, rapid sexual maturation leads to inbreeding and multigenerational coexistence, which in turn affects the germplasm [4,5]. The sex regulation mechanism of *M. nipponense* is currently unclear. Only by understanding this mechanism can we regulate the speed of sexual maturity in *M. nipponense*, thereby solving the problems of yield and germplasm caused by rapid sexual maturity. Therefore, we analyzed the gonadal transcriptome of *M. nipponense* and screened for a significantly expressed gene, *Mn-XRN1*, in the RNA degradation and ribosomal pathways.

**Figure 1:**
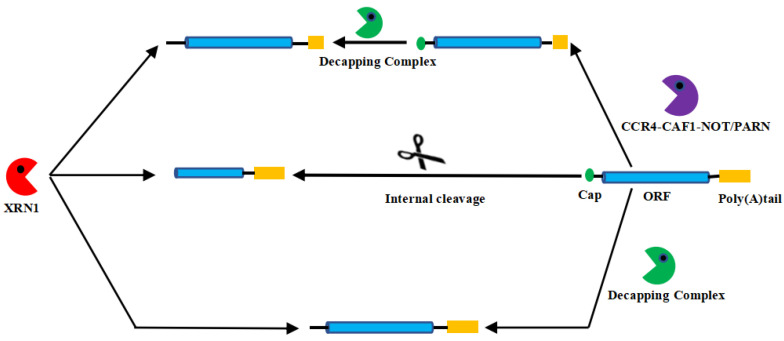
The degradation mechanism of *XRN1* in eukaryotes.

*XRN1* is an important and conserved ribonuclease that degrades RNA in the 5′-3′ direction in the cytoplasm [6]. It is well-known that eukaryotic messenger RNA (mRNA) has two complete stability determinants, 5′7-methylguanosine cap and 3′ poly(A)tail, that protect transcripts from exonucleases and enhance translational initiation. Therefore, in order for degradation to begin, either of these two structures must be destroyed, or the mRNA must undergo internal cleavage by intranuclear dissolution attack [7]. This coincides with the three pathways by which *XRN1* degrades mRNA in eukaryotes (Figure 1). (1) Deenylation-dependent degradation occurs. The mRNA degradation process involves several steps. First, the mRNA is dealkenylated by the CCR4–CAF1–NOT1 complex or PARN enzyme to eliminate most of the 3′poly(A) [8,9]. Then, the 5′ cap is hydrolyzed by a cap-removing complex containing *DCP2*, and, finally, the mRNA is degraded by *XRN1* in the 5′–3′ direction [7,10,11,12,13]. (2) Independent of deenylated degradation, such as nonsense-mediated decline (NMD), occurs. NMD targets and certain long noncoding RNAs can bypass deenylation, directly remove the 5′ cap, and then be degraded by *XRN1* in the 5′–3′ direction [14,15,16]. (3) Nuclear cracking-dependent degradation also occurs. The mRNA is lysed internally, producing unprotected 5′ and 3′ fragments. The 3′ fragments are degraded by *XRN1* [17,18,19]. In addition, *XRN1* is involved in various aspects of RNA metabolism, such as RNA silencing, rRNA maturation, and transcription termination [11,20].

*XRN1* has been well studied in terms of degradation mechanisms. However, the reproduction-related functions of *XRN1* have been poorly studied. In yeast, mutations in *XRN1* lead to larger cells, increased doubling time, and defective spore production [21,22]. Knocking down the *XRN1* gene in *Caenorhabditis elegans* results in a failure of abdominal closure, eventually leading to death of the embryo in the twofold stage of development [23]. *Pacman* is the homologue of *XRN1* in *Drosophila*. Mutations of *Pacman* in *Drosophila* can lead to a variety of developmental phenotypic defects, including decreased fertility, dull wings, and bristle defects [24,25]. *Saccharomyces cerevisiae* cells lacking *Xrn1p* exhibit increased chromosome loss, nucleosome defects, and impaired spindle isolation in cellular processes associated with microtubule function [26]. Furthermore, *XRN1* may be essential for proper bone formation in humans. Mutations in *XRN1* can lead to osteosarcoma, which is produced by mesenchymal-derived cells that are unable to differentiate correctly to produce an unmineralized portion of the bone matrix called osteoids [27,28].

The present study focused on female *M. nipponense,* to determine the potential function of *Mn-XRN1* in the reproduction of *M. nipponense,* and to identify the regulatory relationship with other genes. We used real-time fluorescence quantitative PCR to analyze the expression of *Mn-XRN1* in different tissues, different developmental stages, and different ovarian reproduction stages of *M. nipponense*. In situ hybridization (ISH) was used to determine the location of the *Mn-XRN1* gene. The expression of the *Mn-XRN1* gene was knocked-down using RNA interference (RNAi) technology. Following RNAi, we counted the number of each ovarian stage in *M. nipponense* to observe the effect on ovarian development after *Mn-XRN1* knockdown.

## 2. Materials and Methods

### 2.1. Animals

Healthy female *M. nipponense* of consistent weight required for the present study were obtained from the Freshwater Fisheries Research Center, Chinese Academy of Fishery Sciences (120°13′44″ E, 31°28′22″ N). They were cultured in tanks with circulating water and fed with snail meat twice per day.

### 2.2. Bioinformatics Analysis

The cDNA fragment of the target gene *Mn-XRN1* was obtained from the *M. nipponense* transcriptome cDNA library in our laboratory. The ORF Finder (https://www.ncbi.nlm.nih.gov/orffinder/ (accessed on 20 June 2022)) was used to predict the *Mn-XRN1* open reading frame and the BLAST tool (https://blast.ncbi.nlm.nih.gov/Blast.cgi#alnHdr_317467911 (accessed on 23 June 2022)) was used for comparison to analyze sequence. Based on the known cDNA fragment, we used the Primer-BLAST (https://www.ncbi.nlm.nih.gov/tools/primer-blast/ (accessed on 12 July 2022)) to design the specific primers which were used to verify *Mn-XRN1*. The specific primers are listed in Table 1 and were sent to the Shanghai Exsyn-bio Technology Company for sequencing. The phylogenetic tree was constructed based on amino acid sequence using MEGA5.1 software and the online website iTOL (https://itol.embl.de/ (accessed on 15 August 2022). Multiple sequence alignment of *Mn-XRN1* amino acids was performed by DNAMAN 6.0 software. The three-dimensional protein structure of *Mn-XRN1* was constructed by the online website (https://swissmodel.expasy.org/ (accessed on 17 August 2022). The online program Expasy (https://web.expasy.org/protparam/ (accessed on 20 August 2022)) was used to calculate the protein molecular weight and isoelectric point. The conserved domain of the protein was analyzed by InterpPro (https://www.ebi.ac.uk/interpro/ (accessed on 25 August 2022)).

### 2.3. RNA Extraction and cDNA Synthesis

An RNAiso Plus kit (Takara, Otsu City, Shiga Prefecture, Japan) was used to extract RNA from the whole tissues of the prawns. An electrophoresis apparatus (Bio-Rad, Hercules, CA, USA) was used to detect the quality of the RNA. Reverse transcriptase M-MLV kits (Takara) were used to synthesize the first strand of cDNA.

### 2.4. The qPCR Analysis

The Bio-Rad iCycler iQ5 Real-Time PCR System (Bio-Rad, Hercules, CA, USA) was used to detect the expression levels of genes in different tissues. The reaction system and procedures are referred to in a previous study [29]. All primers used for qRT-PCR are listed in Table 1. The 2^−△△Ct^ method was used to calculate the expression level of all genes required for this study. EIF (eukaryotic translation initiation factor 5A) was used as the reference gene [30].

### 2.5. In Situ Hybridization (ISH)

*M. nipponense* ovarian tissues at five different stages of reproduction were collected. Probes were designed according to known sequences. Three ISH experiments were performed on each tissue to analyze the mRNA locations of *Mn-XRN1*. The detailed steps are described in previous studies [5,29].

### 2.6. RNA Interference (RNAi)

The potential function of *Mn-XRN1* was explored using RNAi. The specific RNAi primers, which are shown in Table 1, were designed by the snap Dragon programs (https://www.flyrnai.org/snapdragon (accessed on 5 September 2022)). The Transcript AidTM T7 High Yield Transcription kit (Fermentas, Inc., Waltham, USA) was used to synthesize the *Mn-XRN1* dsRNA. The *Mn-XRN1* dsRNA was injected into the pericardial cavity. Each prawn was injected with 12 μg/g *Mn-XRN1* dsRNA [31]. On the 5th and 10th day after injection, supplementary injection was performed to maintain the interfering effect [31]. A total of 300 female prawns in the second period (O2 period) of ovarian development were carefully selected and divided into six groups. These groups were divided into three experimental groups and three control groups. The *GFP* dsRNA was injected into control groups.

## 3. Results

### 3.1. Sequence Analysis of Mn-XNR1

**Figure 2:**
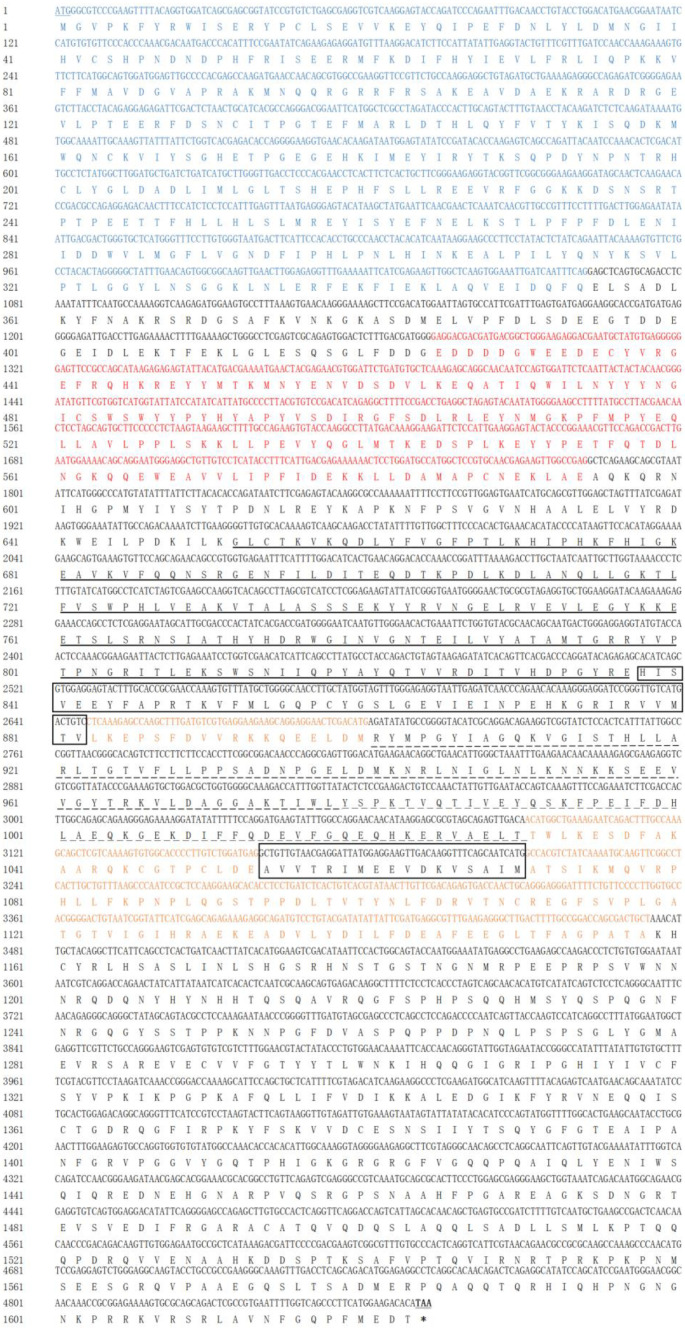
The nucleotide and speculated amino acid sequences of the *Mn-XRN1* gene in *M. nipponense*. The conserved domain CR1 is marked in blue and the conserved domain CR2 is marked in red. Domain D1 is marked with a thick black underline. Domain D2 is framed with a border. Domain D3 is marked with a dashed line. The orange area is domain D4. The starting codon ATG is marked with a blue underline. The stop codon TAG is in bold and marked with a sliding line, and the stop codon TAG in the amino acid sequence is indicated by an asterisk (*) (Supplementary Materials).

The full-length cDNA sequence of *Mn-XRN1* was 12,128 base pairs (bp) long. Its ORF was 4872 bp long and encoded 1623 amino acids. The lengths of the 5′ untranslated region (UTR) and the 3′−UTR were 125 bp and 7131 bp, respectively. The molecular weight and theoretical isoelectric point of the protein were 185.60176 kDa and 6.33, respectively. According to the prediction results, *Mn-XRN1* had two highly conserved regions: CR1 and CR2. The residue orders were 1−354 and 425−594, respectively. Following the CR2 domain, this domain was called D. The D domain was divided into D1, D2, D3, and D4. The residue sequences corresponding to D1 domain and D3 domain were 652−837 and 901−1030, respectively. The D2 domain contained two residue fragments, 837−882 and 1054−1070. Finally, D4 domain included three residue fragments, 883−900, 1031−1053, and 1071−1158 (Figure 2).

**Figure 3:**
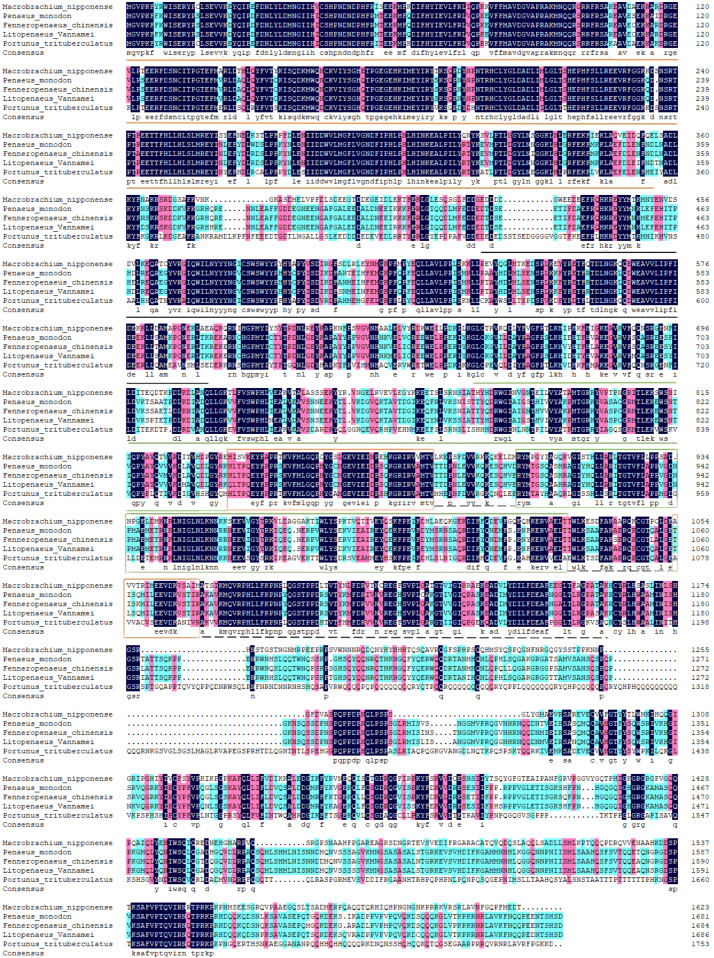
The *Mn-XRN1* predicted amino acid sequence versus the sequence of other species. The predicted amino acid sequence of *Mn-XRN1* in *M. nipponense* (OQ973288) was compared with *XRN1* from *P. monodon* (XP_037803351.1), *F. chinensis* (XP_047471890.1), *L. vannamei* (XP_027206575.1), and *P. trituberculatus* (XP_045106121.1) by the DNAMAN program. CR1 domain, CR2 domain, D1 domain, D2 domain, D3 domain, and D4 domain are marked with orange underline, black underline, green underline, an orange box, a green box, and black dashed lines, respectively.

The results of amino acid sequence comparison between *M. nipponense* and other crustaceans showed that *M. nipponense* had 55.30%, 54.67%, 55.11%, and 53.85% homology with *P. monodon*, *F. chinensis*, *L. vannamei*, and *P. trituberculatus*, respectively (Figure 3).

**Figure 4:**
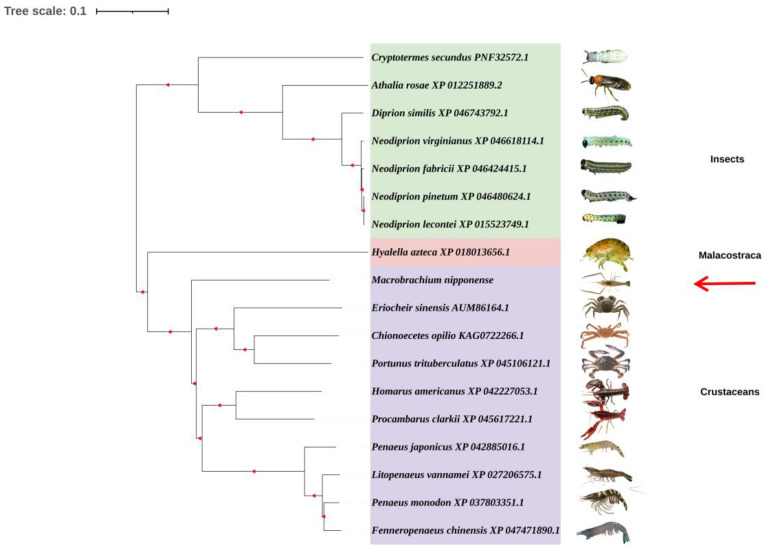
A phylogenetic tree of *XRN1* amino acid sequences. GenBank accession numbers are shown in different colors covering the area. *Mn-XRN1* is marked with a red arrow.

The phylogenetic tree was divided into two main branches. One was an insect, the other was a crustacean, and there was a separate mollusk. *M. nipponense* was one of the crustacean clades (Figure 4).

**Figure 5:**
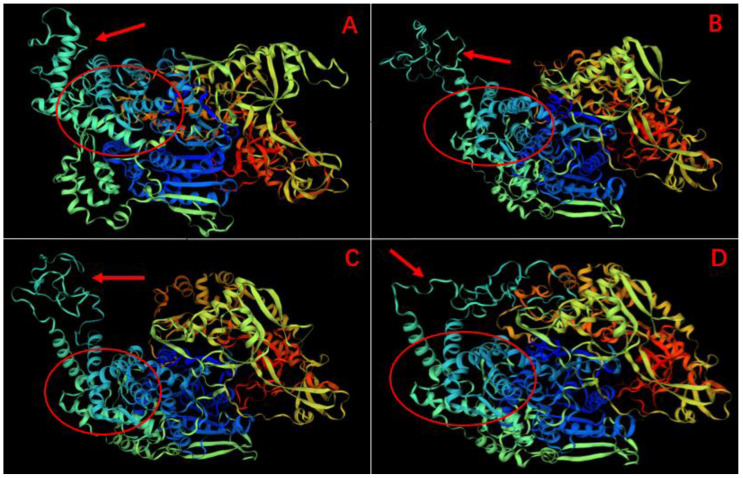
The 3D-structures of *Mn-XRN1* and *XRN1* predicted by SWISS-MODEL. (**A**) displays the 3D-structure of the gene *Mn-XRN1* of *M. nipponense*. (**B**–**D**) display the 3D-structures of the gene *XRN1* in *P. monodon, F. chinensis*, and *L. vannamei*, respectively. The CR1 and CR2 domains are marked with red ellipses. The CR1–CR2 linker is marked with a red arrow.

The three-dimensional protein structure of *Mn-XRN1* compared with the three-dimensional structure of *L. vannamei, P. monodon*, and *F. chinensis* is shown in Figure 5. The results showed that *M. nipponense* and other species shared the same CR1 and CR2 domains. The three-dimensional spatial structure of proteins had high similarity. The CR1 and CR2 domains were connected by a CR1–CR2 linker structure.

### 3.2. Spatial–Temporal Expression Analysis of Mn-XRN1

**Figure 6:**
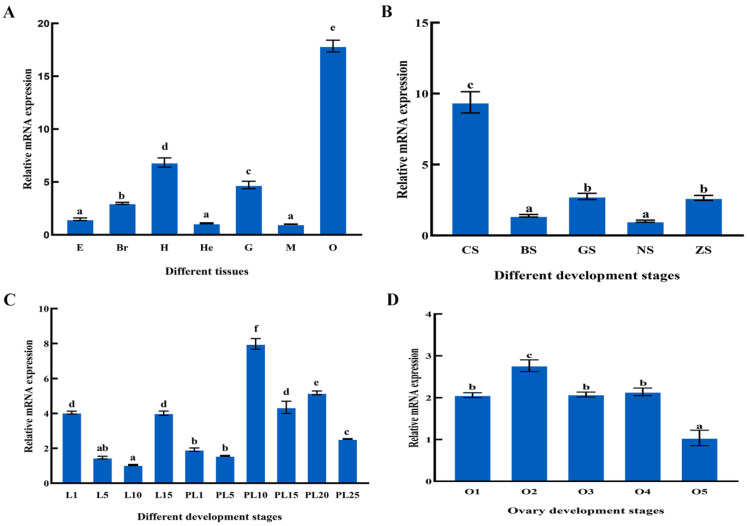
The mRNA expression level of *Mn-XRN1* in the different tissue of *M. nipponense* (**A**). E: eyes, Br: brain, H: heart, He: hepatopancreas, G: gills, M: muscles, O: ovary. The mRNA expression level of *Mn-XRN1* in the different periods of embryos (**B**). CS: cleavage stage, BS: blastula stage, GS: gastrula stage, NS: nauplius stage, ZS: zoea stage. Expression modes of *Mn-XRN1* at different stages of ontogeny (**C**). L1: the 1st-day after hatching, PL1: the 1st-day after larvae, and so on. The mRNA expression level of *Mn-XRN1* in the different reproduction periods of the ovaries (**D**). O1: undeveloped period, O2: developing period, O3: nearly ripe period, O4: ripe period, O5: spent period. Statistical analysis was carried out using the one-way ANOVA method. Data are shown as mean ± SEM(*n* = 6). Bars marked with different letters represent significant differences in data (*p* < 0.05).

At the organizational level, we analyzed the expression of *Mn-XRN1* in different tissues of *M. nipponense* (Figure 6A). The highest expression of *Mn-XRN1* occurred in the ovaries, followed by the heart. *Mn-XRN1* was least expressed in muscle. The expression of *Mn-XRN1* in the ovaries and muscles differed by 18-fold (*p* < 0.05).

Next, the expression modes of *Mn-XRN1* at different embryonic and metamorphic developmental stages were examined. The embryonic period starts from early oogenesis to the pre-hatch stage. During embryonic development, maximum expression of *Mn-XRN1* in the CS stage was observed (Figure 6B). *Mn-XRN1* mRNA levels then decreased significantly after the CS stage and remained at a low level (*p* < 0.05).

The metamorphic developmental period begins at the L1 stage and ends at the PL25 stage. The expression of *Mn-XRN1* showed a downward trend from L1 to L10 stages (Figure 6C). At the L15 stage, the expression increased suddenly and significantly. In PL10 to PL25 stages, the expression of *Mn-XRN1* was higher than that of the larval development stages and reached a maximum in the PL10 stage (*p* < 0.05).

The mRNA expression level of *Mn-XRN1* in the ovary showed a trend of first increasing and then decreasing, and reached a maximum in O2 stage (Figure 6D). The mRNA expression of *Mn-XRN1* in the O1, O3, and O4 stages was not much different, but it decreased significantly in the O5 stage (*p* < 0.05).

### 3.3. Localization of Mn-XRN1 in the Ovaries

**Figure 7:**
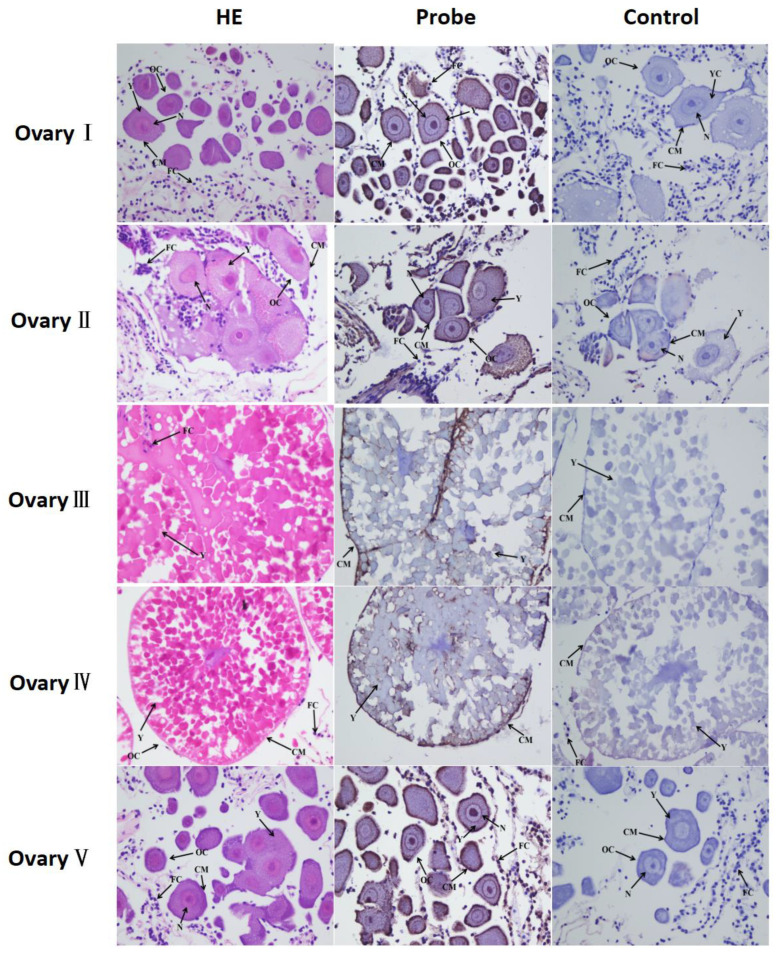
Histological section of various ovary periods in *M. nipponense*. OC: oocyte; N: nucleus; CM: cytoplasmic membrane; Y: yolk granule; scale bars: ×400.

In situ hybridization was used to detect the location of *Mn-XRN1* mRNA in different stages of the ovary (Figure 7). The ISH results showed that the signal of *Mn-XRN1* was stronger in the yolk granules and follicular cells. More signals were gathered in the nucleus in the O2 period. *Mn-XRN1* signals were also detected in the cytoplasmic membrane and nucleus of oocytes.

### 3.4. Effects of Mn-XRN1 Gene Silencing after RNAi on Ovarian Reproduction

**Figure 8:**
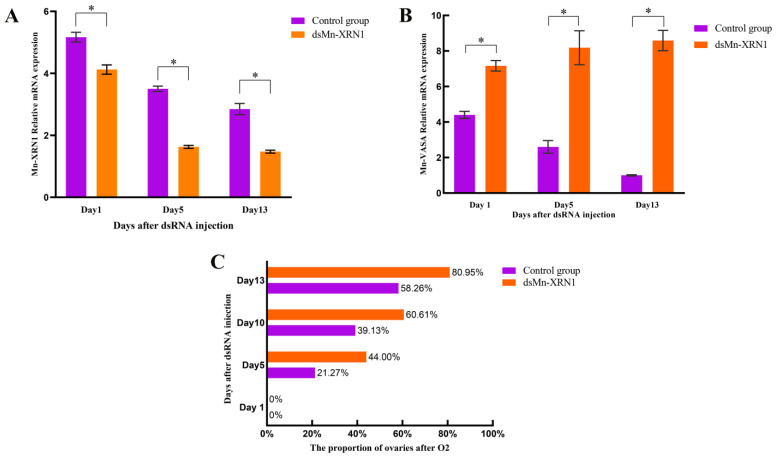
The mRNA expression levels of *Mn-XRN1* and *Mn-VASA* after RNAi. (**A**) *Mn-XRN1*; (**B**) *Mn-VASA*. Data are shown as mean ± SEM. The significance was tested with Student’s *t*-test (*n* = 6) and differences were considered significant when *p* < 0.05 (*). On different days after injection, a proportion of ovaries developed more than the second stage of *M. nipponense* (**C**).

Based on the previous expression pattern of the *Mn-XRN1* gene in different tissues, we used RNAi to determine the function of the *Mn-XRN1* gene. On the 1st, 5th, and 13th days after injection, the interference efficiency reached 20.24%, 53.51%, and 48.11%, respectively (Figure 8A). After RNAi, the proportion of ovarian maturity after the O2 phase was greater than that of the control group at the same periods (Figure 8B). The interference efficiency results were consistent with the observation of ovarian maturation. There were significant differences between the experimental and control groups.

After RNAi silenced *Mn-XRN1*, we found a significant increase in the expression of *Mn-VASA*, a member of the Dead Box family, in the ribosomal pathway. Compared with the control group, the differences in *Mn-VASA* gene expression on days 1, 5, and 13 in the experimental group were 62.78%, 214.99%, and 754.37%, respectively (Figure 8C). The expression of *Mn-VASA* in the control group gradually decreased with the reproduction of the ovaries.

## 4. Discussion

*XRN1* has a single active site with a narrow inlet that removes secondary structures as the RNA crosses the gap [32]. The 5′ orientation of *XRN1* has a series of conservative domains: CR1, CR2, and D. The D Domain can be divided into D1, D2, D3, and D4 [32,33]. These conserved domains have been found in *XRN1* of yeast, *C. elegans*, mice, and other creatures, and have high homology. They play an important role in *XRN1*’s correct degradation of various RNA substrates [34]. Domain CR1 contains seven strictly conserved acidic residues that coordinate metal ions for catalysis; domain CR2 restricts access to the active site, ensuring that *XRN1* is the only exoribonuclease; domain D1 is essential for *XRN1* nuclease activity and may play an active role in determining or stabilizing the conformation of the N-terminal fragment. This may be a key factor in *XRN1* catalysis; domains D2–D4 may have an impact in ensuring the correct conformation of domain D1, thereby indirectly enhancing the stability of the N-terminal fragment conformation [32]. In the present research, we obtained and verified the sequence of *Mn-XRN1* from *M. nipponense* for the first time, and compared the amino acid sequence with other species. The phylogenetic tree and amino acid sequence alignment results showed that *M. nipponense* had high homology with crustaceans and was more conserved than other crustaceans. This dovetails with the trend revealed by our previous findings. Compared to other crustaceans, *M. nipponense* appeared earlier in evolution and had a more conserved genome [35].

We used qRT-PCR to analyze the expression of *Mn-XRN1* in *M. nipponense*. At the tissue level, *Mn-XRN1* was significantly expressed in the ovaries. This result showed that *Mn-XRN1* might play a key role in the regulation of ovarian reproduction. During the embryonic stage, highly expressed genes directly participate in embryonic development or prepare for future physiology [36]. The expression of *Mn-XRN1* peaked at the cleavage stage of embryonic development. This suggested that *Mn-XRN1* might be involved in the process of oocyte mitosis. L1 is the first day after hatching, and L15 is the critical day of metamorphosis before the post-larval developmental stages of *M. nipponense* [37,38]. The relatively high expression of L1 and L15 meant that *Mn-XRN1* was associated with post-membrane development and metamorphosis. The post-larval developmental periods have been shown to be a critical period for gonad differentiation and development in *M. nipponense*. According to histological observations, *M. nipponense* began to develop gonadal primordium at PL10. After PL10, the gonads began to differentiate and develop [39]. The mRNA expression level of *Mn-XRN1* in the PL10–PL20 stage was higher than that in the previous stage and reached a maximum in the PL10 stage, indicating that *Mn-XRN1* might be involved in activating and promoting the gonadal differentiation and development in *M. nipponense.*

Ovarian reproduction in *M. nipponense* can be divided into five stages: O1, undeveloped stage; O2, developmental stage; O3, near mature stage; O4, mature stage; and O5, declining stage [3]. The mRNA expression of *Mn-XRN1* reaches the maximum at O2 stage. These results indicated that *Mn-XRN1* may be associated with yolk deposition. In Drosophila, *Pacman* is also involved in eggogenesis of female nurse cells and within the yolk [40]. On the other hand, the follicular cavity derived from follicular cells is formed in O2 stage [5]. One plausible explanation is that *Mn-XRN1* may be involved in activating ovarian development, especially follicle production. ISH results showed detection of *Mn-XRN1* signaling in oocytes and follicles of O1, O2, and O5 stages. The ISH result provided a new basis for the viewpoint that *Mn-XRN1* may be involved in ovarian maturation and follicle formation in *M. nipponense.*

RNAi is a technology that inhibits gene expression by using short double-stranded RNA molecules, and is widely used in gene function research in *M. nipponense* [41,42,43,44,45]. We used RNAi technology to further explore the functionality of *Mn-XRN1*. On the 1st, 5th, and 13th days after RNAi with *Mn-XRN1*, the expression of *Mn-XRN1* in the ovary of the experimental group decreased significantly compared with the control group. This indicated that *Mn-XRN1* dsRNA can effectively inhibit the expression of *Mn-XRN1* in this study. Initially, the ovary of *M. nipponense* in the experimental group and the control group was in the O2 stage. On the 5th, 10th, and 13th days after dsRNA injection, the proportion of ovaries developing to the stage after O2 was greater than that of the control group. The results showed that, after *Mn-XRN1* silencing, the ovarian reproduction of *M. nipponense* was significantly accelerated. Therefore, *Mn-XRN1* had an inhibitory effect on ovarian reproduction. In the search for regulatory relationships with other related genes, we found that the gene *Mn-VASA* was significantly regulated by *Mn-XRN1* in the ribosome genesis pathway. After silencing of the *Mn-XRN1* gene, the expression of *Mn-VASA* gene increased significantly, indicating that *Mn-VASA* was regulated by negative feedback from *Mn-XRN1*. *VASA* is thought to be involved in the assembly and transport of vitellin mRNA in follicular cells during secondary yolk genesis [46]. Based on the existing research results, we propose that in *M. nipponense*, *Mn-XRN1* inhibits the production of vitellinogen by inhibiting the expression of the gene *Mn-VASA*, which is responsible for the production and transport of vitellinogen, thereby controlling development of the ovaries.

## 5. Conclusions

In summary, we identified the 5′−3′ direction ribonuclease exonuclease gene *Mn-XRN1* in *M. nipponense*. The results of this study indicated that *Mn-XRN1* was associated with early physical changes in *M. nipponense*, such as post-membrane development and metamorphosis. It may also play an important role in gonadal differentiation. In terms of ovarian reproduction, we found that *Mn-XRN1* was associated with yolk deposition and follicle production. Further RNAi results showed that *Mn-XRN1* inhibited ovarian development. More importantly, we found that the gene *Mn-VASA* was strongly inhibited by *Mn-XRN1*. *Mn-VASA* was associated with the synthesis of vitellinogen. Finally, we proposed a viewpoint that *Mn-XRN1* might inhibit the production of vitellinogen by inhibiting the expression of *Mn-VASA*, thereby controlling ovarian maturation. This study enriched the understanding of molecular mechanism of female sexual maturation during the breeding period of *M. nipponense* and provided new insights for studying sexual maturity in crustaceans.

## Figures and Tables

**Table 1:** Primers designed in this study.

Primer Name	Length (bp)	Primer Sequence (5′–3′) Forward/Reverse	Usage
*Mn-XRN1*-V1	1017	AAGGAGGCTGTAGATGCTGAAAA AATACTCTCTCTTATGCTGGCGG	For *Mn-XRN1* verify
*Mn-XRN1*-V2	624	GTATCCGTGTCTGAGCGAGGTC AAGTGAGGTTCGTGGGAGGTC	For *Mn-XRN1* verify
*Mn-XRN1*-V3	640	ATCGGGGAGAAGTCTTACCTACAG CCACTGTTCAAATAGCCCCCT	For *Mn-XRN1* verify
*Mn-XRN1*-V4	620	ATATAATTGACGACTGGGTGCTC TGACCACGAACATATCCCG	For *Mn-XRN1* verify
*Mn-XRN1*-V5	659	AAGAGTTCCGCCAGCATAAGAGA GCACAACCCCTTCAAGATTTT	For *Mn-XRN1* verify
*Mn-XRN1*-V6	412	CTGTTGTCCTCATACCTTTCATTG GGTTTGGTGTCCTGTTCAGTGATG	For *Mn-XRN1* verify
*Mn-XRN1*-V7	270	TGCACCGCGAACCAAAGT TTGTCCGCCGAAGGTGG	For *Mn-XRN1* verify
*Mn-XRN1*-V8	363	ACACATACCCCATAAGTTCCACA GTTGCGTACACCAGAATTTCAGT	For *Mn-XRN1* verify
*Mn-XRN1*-V9	338	GAGGTGCTGGAAGGATACAAGAA TCTCCCAAACTACCATAGCAAGG	For *Mn-XRN1* verify
*Mn-XRN1*-V10	397	ACAGTCTTCCTTCTTCCACCTTC TTACAACAGCCTCATCCAGACAA	For *Mn-XRN1* verify
*Mn-XRN1*-V11	380	CAGAGCAGAAGGGAGAAAAGGAT ATGAATACCGATTACAGTCCCCG	For *Mn-XRN1* verify
*Mn-XRN1*-V12	238	CGGGGACTGTAATCGGTATTCAT TTATTCCACACAGAGGGTCTTGG	For *Mn-XRN1* verify
*Mn-XRN1*-V13	245	CCAAGACCCTCTGTGTGGAATAA GACTTGGTAACTGATTGGGGTCT	For *Mn-XRN1* verify
*Mn-XRN1*-V14	408	AGACCCCAATCAGTTACCAAGTC CAGGTATTGCTTCAGTGCCAAAA	For *Mn-XRN1* verify
*Mn-XRN1*-V15	483	TTTTGGCACTGAAGCAATACCTG TCTGTTACGAATGACCTGAGTGG	For *Mn-XRN1* verify
*Mn-XRN1*-V16	231	CCACTCAGGTCATTCGTAACAGA CATGAAGGGCTGACCAAAATTCA	For *Mn-XRN1* verify
*Mn-XRN1*-q	238	CGGGGACTGTAATCGGTATTCAT TTATTCCACACAGAGGGTCTTGG	For RT-qPCR
*Mn-XRN1*-d	352	TAATACGACTCACTATAGGGCACTGAAGCAATACCTGCGA TAATACGACTCACTATAGGGAAGATCGGCACTCAGCTGT	For *Mn-XRN1* dsRNA
*GFP*-d	533	TAATACGACTCACTATAGGGAGTGGAGAGGGTGAAGG TAATACGACTCAC TAATACGACTCACTATAGGGAGGGCAGATTGTGTGGAC	For *GFP* dsRNA
*EIF*	179	CATGGATGTACCTGTGGTGAAAC CTGTCAGCAGAAGGTCCTCATTA	For RT-qPCR
*Mn-XRN1* sense Probe		TGGATTGTAATCTGGCTGACTCTTGGTGTA	For *Mn-XRN1* ISH analysis
*Mn-XRN1* anti-sense Probe		TACACCAAGAGTCAGCCAGATTACAATCCA	For *Mn-XRN1* ISH analysis

## Data Availability

All data are available in the manuscript.

## Supplementary Materials

The following supporting information can be downloaded at: https://www.mdpi.com/article/10.3390/genes14071454/s1.
