# Supplementary material for: Mn-XRN1 Has an Inhibitory Effect on Ovarian Reproduction in Macrobrachium nipponense
Source: Genes (Basel). 2023 Jul 16;14(7):1454. doi: 10.3390/genes14071454 (PMC10380074; doi:10.3390/genes14071454)
Supplement: Supplementary file 1 [file genes-14-01454-s001.zip › Full length sequence.pdf]

> ***Macrobrachium nipponense XRN1***

CACCAGGTCTGGGAATGGCACCCGTCCCGAACGCAATAGTAAATTGAATCAGTGC
AACATCAACTGGACTTGTATGGCGAAGGAATTGTCACTTACAGAACCAGTGAACC
ACCAGGATACACAAAATGGGCGTCCCGAAGTTTTACAGGTGGATCAGCGAGCGGT
ATCCGTGTCTGAGCGAGGTCGTCAAGGAGTACCAGATCCCAGAATTTGACAACCT
GTACCTGGACATGAACGGAATAATCCATGTGTGTTCCCACCCAAACGACAATGACC
CACATTTCCGAATATCAGAAGAGAGGATGTTTAAGGACATCTTCCATTATATTGAGG
TACTGTTTCGTTTGATCCAACCAAAGAAAGTGTTCTTCATGGCAGTGGATGGAGTT
GCCCCACGAGCCAAGATGAACCAACAGCGTGGCCGAAGGTTCCGTTCTGCCAAG
GAGGCTGTAGATGCTGAAAAGAGGGCCAGAGATCGGGGAGAAGTCTTACCTACA
GAGGAGAGATTCGACTCTAACTGCATCACGCCAGGGACGGAATTCATGGCTCGCC
TAGATACCCACTTGCAGTACTTTGTAACCTACAAGATCTCTCAAGATAAAATGTGGC
AAAATTGCAAAGTTATTTATTCTGGTCACGAGACACCAGGGGAAGGTGAACACAA
GATAATGGAGTATATCCGATACACCAAGAGTCAGCCAGATTACAATCCAAACACTC
GACATTGCCTCTATGGCTTGGATGCTGATCTGATCATGCTTGGGTTGACCTCCCACG
AACCTCACTTCTCACTGCTTCGGGAAGAGGTACGGTTCGGCGGGAAGAAGGATAG
CAACTCAAGAACACCGACGCCAGAGGAGACAACTTTCCATCTCCTCCATTTGAGT
TTAATGAGGGAGTACATAAGCTATGAATTCAACGAACTCAAATCAACGTTGCCGTT
TCCTTTTGACTTGGAGAATATAATTGACGACTGGGTGCTCATGGGTTTCCTTGTGGG
TAATGACTTCATTCCACACCTGCCCAACCTACACATCAATAAGGAAGCCCTTCCTAT
ACTCTATCAGAATTACAAAAGTGTTCTGCCTACACTAGGGGGCTATTTGAACAGTG
GCGGCAAGTTGAACTTGGAGAGGTTTGAAAAATTCATCGAGAAGTTGGCTCAAGT
GGAAATTGATCAATTTCAGGAGCTCAGTGCAGACCTCAAATATTTCAATGCCAAAA
GGTCAAGAGATGGAAGTGCCTTTAAAGTGAACAAGGGAAAAGCTTCCGACATGG
AATTAGTGCCATTCGATTTGAGTGATGAGGAAGGCACCGATGATGAGGGGGAGATT
GACCTTGAGAAAACTTTTGAAAAGCTGGGCCTCGAGTCGCAGAGTGGACTCTTTG
ACGATGGGGAGGACGACGATGACGGCTGGGAAGAGGACGAAGAGTTCCGCCAGC
ATAAGAGAGAGTATTACATGACGAAAATGAACTACGAGAACGTGGATTCTGATGTG
CTCAAAGAGCAGGCAACATGCTATGTGAGGGGGATCCAGTGGATTCTCAATTACTA
CTACAACGGGATATGTTCGTGGTCATGGTATTATCCATATCATTATGCCCCTTACGTG
TCCGACATCAGAGGCTTTTCCGACCTGAGGCTAGAGTACAATATGGGGAAGCCTTT
TATGCCTTACGAACAACTCCTAGCAGTGCTTCCCCCTCTAAGTAAGAAGCTTTTGC
CAGAAGTGTACCAAGGCCTTATGACAAAGGAAGATTCTCCATTGAAGGAGTACTA
CCCGGAAACGTTCCAGACCGACTTGAATGGAAAACAGCAGGAATGGGAGGCTGT
TGTCCTCATACCTTTCATTGACGAGAAAAAACTCCTGGATGCCATGGCTCCGTGCA
ACGAGAAGTTGGCCGAGGCTCAGAAGCAGCGTAATATTCATGGGCCCATGTATATT
TATTCTTACACACCAGATAATCTTCGAGAGTACAAGGCGCCAAAAAATTTTCCTTC
CGTTGGAGTGAATCATGCAGCGTTGGAGCTAGTTTATCGAGATAAGTGGGAAATAT
TGCCAGACAAAATCTTGAAGGGGTTGTGCACAAAAGTCAAGCAAGACCTATATTT
TGTTGGCTTTCCCACACTGAAACACATACCCCATAAGTTCCACATAGGAAAAGAAG
CAGTGAAAGTGTTCCAGCAGAACAGCCGTGGTGAGAATTTCATTTTGGACATCAC
TGAACAGGACACCAAACCGGATTTAAAAGACCTTGCTAATCAATTGCTTGGTAAA
ACCCTCTTTGTATCATGGCCTCATCTAGTCGAAGCCAAGGTCACAGCCTTAGCGTC

ATCCTCGGAGAAGTATTATCGGGTGAATGGGGAACTGCGCGTAGAGGTGCTGGAA
GGATACAAGAAAGAGGAAACCAGCCTCTCGAGGAATAGCATTGCGACCCACTATC
ACGACCGATGGGGAATCAATGTTGGGAACACTGAAATTCTGGTGTACGCAACAGC
AATGACTGGGAGGAGGTATGTACCAACTCCAAACGGAAGAATTACTCTTGAGAAA
TCCTGGTCGAACATCATTCAGCCTTATGCCTACCAGACTGTAGTAAGAGATATCACA
GTTCACGACCCAGGATACAGAGAGCACATCAGCGTGGAGGAGTACTTTGCACCGC
GAACCAAAGTGTTTATGCTGGGGCAACCTTGCTATGGTAGTTTGGGAGAGGTAATT
GAGATCAACCCAGAACACAAAGGGAGGATCCGGGTTGTCATGACTGTCCTCAAAG
AGCCAAGCTTTGATGTCGTGAGGAAGAAGCAGGAGGAACTCGACATGAGATATAT
GCCGGGGTACATCGCAGGACAGAAGGTCGGTATCTCCACTCATTTATTGGCCCGGT
TAACGGGCACAGTCTTCCTTCTTCCACCTTCGGCGGACAACCCAGGCGAGTTGGA
CATGAAGAACAGGCTGAACATTGGGCTAAATTTGAAGAACAACAAAAAGAGCGA
AGAGGTCGTCGGTTATACCCGAAAAGTGCTGGACGCTGGTGGGGCAAAGACCATT
TGGTTATACTCTCCGAAGACTGTCCAAACTATTGTTGAATACCAGTCAAAGTTTCC
AGAAATCTTCGACCACTTGGCAGAGCAGAAGGGAGAAAAGGATATATTTTTCCAG
GATGAAGTATTTGGCCAGGAACAACATAAGGAGCGCGTAGCAGAGTTGACAACAT
GGCTGAAAGAATCAGACTTTGCCAAAGCAGCTCGTCAAAAGTGTGGCACCCCTTG
TCTGGATGAGGCTGTTGTAACGAGGATTATGGAGGAAGTTGACAAGGTTTCAGCA
ATCATGGCCACGTCTATCAAAATGCAAGTTCGGCCTCACTTGCTGTTTAAGCCCAA
TCCGCTCCAAGGAAGCACACCTCCTGATCTCACTGTCACGTATAACTTGTTCGACA
GAGTGACCAACTGCAGGGAGGGATTTCTGTTCCCCTTGGTGCCACGGGGACTGT
AATCGGTATTCATCGAGCAGAGAAAGAGGCAGATGTCCTGTACGATATATTATTCGA
TGAGGCGTTTGAAGAGGGCTTGACTTTTGCCGGACCAGCGACTGCTAAACATTGC
TACAGGCTTCATTCAGCCTCACTGATCAACTTATCACATGGAAGTCGACATAATTCC
ACTGGCAGTACCAATGGAAATATGAGGCCTGAAGAGCCAAGACCCTCTGTGTGGA
ATAATAATCGTCAGGACCAGAACTATCATTATAATCATCACACTCAATCGCAAGCAG
TGAGACAAGGCTTTTCTCCTCACCCTAGTCAGCAACACATGTCATATCAGTCTCCT
CAGGGCAATTTCAACAGAGGGCAGGGCTATAGCAGTACGCCTCCAAAGAATAACC
CGGGGTTTGATGTAGCGAGCCCTCAGCCTCCAGACCCCAATCAGTTACCAAGTCC
ATCAGGCCTTTATGGAATGGCTGAGGTTCGTTCTGCCAGGGAAGTCGAGTGTGTCG
TCTTTGGAACGTACTATACCCTGTGGAACAAAATTCACCAACAGGGTATTGGTAGA
ATACCGGGCCATATTTATATTGTGTGCTTTTCGTACGTTCCTAAGATCAAACCGGGA
CCAAAAGCATTCCAGCTGCTCATTTTCGTAGACATCAAGAAGGCCCTCGAAGATGG
CATCAAGTTTTACAGAGTCAATGAACAGCAAATATCCTGCACTGGAGACAGGCAG
GGTTTCATCCGTCCTAAGTACTTCAGTAAGGTTGTAGATTGTGAAAGTAATAGTATT
ATATACACATCCCAGTATGGTTTTGGCACTGAAGCAATACCTGCGAACTTTGGAAG
AGTGCCAGGTGGTGTGTATGGCCAAACACCACACATTGGCAAAGGTAGGGGAAG
AGGCTTCGTAGGGCAACAGCCTCAGGCAATTCAGTTGTACGAAAATATTTGGTCAC
AGATCCAACGGGAAGATAACGAGCACGGAAACGCACGGCCTGTTCAGAGTCGAG
GGCCGTCAAATGCAGCGCACTTCCCTGGAGCGAGGGAAGCTGGTAAATCAGACAA
TGGCAGAACGGAGGTGTCAGTGGAGGACATATTCAGGGGAGCCAGAGCTTGTGCC
ACTCAGGTTCAGGACCAGTCATTAGCACAACAGCTGAGTGCCGATCTTTTGTCAAT
GCTGAAGCCGACTCAACAACAACCCGACAGACAAGTTGTGGAGAATGCCGCTCAT
AAAGACGATTCCCCGACGAAGTCGGCGTTTGTGCCCACTCAGGTCATTCGTAACA

GAACGCCGCGCAAGCCAAAGCCCAACATGTCCGAGGAGTCTGGGAGGCAAGTAC
CTGCCGCCGAAGGGCAAAGTTTGACCTCAGCAGACATGGAGAGGCCTCAGGCAC
AACAGACTCAGAGGCATATCCAGCATCCGAATGGGAACGGCAACAAACCGCGGA
GAAAAGTGCGCAGCAGACTCGCCGTGAATTTTGGTCAGCCCTTCATGGAAGACAC
ATAAAGTGTGAAGTTGTTCACAAATGGTTTTATAAGTGTGATTATGTAATCGGTGTA
AACTACTGACATGTAGAAAAAGATATTTTTGTGTGGATGACTTTATTTTAAATAACA
GAAATACAACGAGATCCTTCATATTGAAGTCCATATTTCATATAATTAGGAAACCCC
CAAGGCCTCGGAGCAGAGAAATAGTTAATGTTTCTAGGAAAGTTTAAGCTTAGATT
TTATTGTTAAATAATGTACAAGAGCATCAGTAAGCAGCAGTATGTAGGAGATGGCA
AGTGTCACACTGCCAAGTGTTTTAATTGGACTGGCTTTTACTCTTTCCCATATTTCA
AGAATTTTGTGGCTTTCAAGTCAGTGCATTCTTGAAATGTGTAATTTTCACCCATGA
AGTGCAAAAGTGTCTTTTTTTCTGTTTTTTATTTTAACTTTGTGGTGCTGATGTTCTA
GATTAATTTTTCTTCTAATTAAAGTAATGTGTGTAGGAGGTGAGTTTCTATATTGTGA
CCATGGTCATTATCGGATGCTTGTGACCAAGGTTAACAAGGCATTGATCTCAAGTA
CCTTCTTGCAGTTAGACGACAATCTACATTCTATATTCTTGACTGTCCCTAAGAACA
AAGTCACAGATATGACATTATGCAAGATGCATTATATAACATTAGTTTGGATAGCTAT
GGAAGTTTGTGTAGTTGGAATGTAGATACACATATCAAATGCTATCTGAAACATATG
TTTACAAAAGTATGCAGCCAGGAGGAGGAAGAGACGTATGATTACCTTTCCAGAA
CCTACTTCTCCAACTCTATTTCATTGTTGATACTCATGACAGTTTCTCTACATTCTAG
TGGGGAAGTTTTTCAAGTCTGAATTGGGAGTTATTTATTTTGGAAAATGTAAACATT
TATAAGAAATATCTATGAATCAATCTTATATTTTTAAAATGTTGCATGGAGCTACAAA
TGGCTTGACAGAGTATGCACGTTATAATTGTTTTCAGCTACATTTGTTTCATTTTGTA
ACTTCCCAAGGACTGAGCTGCAAGGCTGAAATTTCCCATCGTTGTTCAGAATTCAT
GATACTATATTTCAGATTTACAGTATTCTTTACTGTAAAAGTAAAGTACAGGGGAAA
CATGCAATATCATAATTGTATTAAGAAAATGTTTTAACCCTAGCTACAATGAATAGAT
ATACTGGTTTTCATGAGATTTTTATAATTCTTATCACTTACTGCAGTAGCCATGTCAC
TTATTTTATATTAATGTGTCATGTTGGTATTTCTTGCTGAAAGAAATATCTATAAACCC
CACAGCACTGCCATTTTCACTTTAATAGATGTATTTAACTTGTGAAAATAAAATCCC
CTGCATTGAACATTGTAATTGTAGTGTTCATTGTTCTATATAACCAGAACTTGTACA
ATCCCCCATGAGTGTCACAGCAGGTCTTGAGTGAAATATTTGAGATTTAAAGAAGG
AACTATGCAATACTCTAGACTCATAGGTCTGGATAAACTAATCCTTATAATAATAATA
GTATACTGATGGTTTCTAATCTCCCTTTTGGTGTGAGAATTGTTGTTCTTTCTTCTTA
GTGAAACTAGTCCATTCTTGTAACTCTCTAATGCCTTTTTGTTTTCACAGCAGATCT
CTACAGTAATCTCAGCAGATTGAGATATAAAATGTGGACCCCAACAAAAATGTTAA
AATTCAGTCTTTTGAAATGCTTTGGACGCTCCTTTGAATTTATCCACCTTTAGTTCTT
TCATATATGGTAGGATTTTTTCATGAATTACGTTAAGTTTGTGTAAATTTGGAATCAT
TTTAATTTTCGTCCTTCTCAGTTAGGGCTCTTAAAAGATAACTCATATAAACATGTAC
CTGTACTGAATCAGATTTACCTGTTTGTGTAGGTGATTTTTCAGTATTTTAATGTTTT
GTTTTGCAATCATATGTTCATCTTGGTAATTGAGAATGAGTTAGAATTTTGTACTTGT
TTTGGTCATTTCATGGTTAGCAAATAACCCTACATAAAGCAATGTTTTTCCTGTGAG
TGAATGTTACAGTTGAATGTGCTATTTTAAATAGTATGGCCAATTTGGGATAATAAAT
GTTCAGCACAAAAAGAATTATGAGAGAGAGAGAGAGAGAAAATGCAAAGGGTAG
AAATAAATAGAACATAGTACGTAGCAAGAGAAGAAGATGAATGGAAGTACTGATG
AGGAAATTATCAAACAAAAGATTGACATTGATTGATGCATCAAGATTACAGCGCCA

CCTATTACCAACTTCTGATTTTGAACAACCATCCCTTTTTGGTTCAAATAATTTCTT
CTTCTGTGGAGAGGGGCTGGGTGATCAATACATTCCAGAAACTTTTTCTAGTTAGT
CGCATTTCTTGGGAATGTCATTCGTGTTATTGGACTAACAACAATTACGATAACTTG
TCACGTACCAGCAGCCAATTCAACAACATTATCTACATGGATGGGTGACATACATTA
CCAACAAATGTAAAAATAATTCTGGAATATAGAATTACAGAAACATATACAGTATAT
GTATATGAGTAAATAGTAAGTACATAGTAAATAGTACATATGACTACACTTGATACTT
TCAGTACTTAATCATTCATAAATATAGACATGTAGATGTTTCCTGGCCTACGTTGACT
CGTTTTATATTTCAGTTTTACAGTGGTAGAAACTGTATTCTGAACTTTGAATTTTCAT
TTTGTCTCAAATATGTAATTGGATGTTTGCCCAGTCTGGGAAATATTTTGGGGAAAC
CTGGGTGGTTGCTCTGTCCCCAGCCATTTGGAGAACTTAATAGTTCACAGGTAATG
AGAAATGGTGCCTTGCGGTACCCTGCTATCTTCCAGAGATGTCTTCAAGAAGTTCT
GAGACCATTCTCCTTTGGACCTGTGGTGACTGCTCTCTATAGGTTATGTAGTTTCCA
AGTTCCTCTCGGACATAAGTGACTCGGATGACCCAAGGCTAGAGACTGAATGGAT
GAGTGACTGGCTCTCTTTCTGTTGCTCTTCTTCCTTCCTTTGCAAGAGTGTAATCAG
GATCACTACATGCTGGGCAGGCCAAGTTGCAGATATACTTCACAATTGAGCATCTT
AACTTAGAGCTCTGGTTCACAAAGGGTGACTCCCTCCACTCTCCCTAAAGCTATTT
TCTTAAGCCATCAATTTATGATTGACCCAGATACTTGCTGTCCGCTGATCTGGGCTA
CTGTGTTGTCAGGGCAACTCTGGTTCTCAGAAGTTTTCCAAGCGTGAGAGTTCAG
ATGCATCTCACTCTGGCTTAGACCTAATCAATTTTGCATCCCTATGCCAGATTGTATG
GACAATTGCTGGAATTATTGCTCGTGAGCACACAATCGTGACCAGGGACAGCAATT
CTGAGTGGAAATTCAACCTACCAGTCAGTTGAGAGATGCTCTTTCCACCTAAGAAT
GAGTCTTTTACGAGAAAGGCTGTGGTTTATAGCCCTAGCATTTGATCGTAATCCCAT
TTCAACTACCCAGCATAGTCCCTGATGCCGAACAAAATATGCGTGGTGACGGCAAG
TGAGTGGAACTCCTTCCCCCCTTCCAATCACTGCCCCGCTTATCACTTGGACCACC
AAGTGAGCTACCTTGTTACCAAGATTCCATGACCTATTCCAGCTTGTACTACTAAAA
GCTACTCCTATATGTAAGGCTCTTGTTTGTATACTAGGAAAAGTATAACTTGTTATAA
AAATGAAATTTTTCAGCTTTTCAACTTGAAACATGAATTTACCATGTTGAAGTATCC
ACTCAGCCAATTTTTTAATGTGTTTTTGGTAGCTGGAACAGTTGAAATGAAAAGAA
TGAGTTTGAAATTGTCCTTAGCATTTTCATATGTTTTTTGGACACAAACAAACATAC
ATTGTTAACATGTGACTTTCCTCTAAGGAGTGATCAGTAAAAGGTGAATTACAATG
TGAAGTTGACTCCAAGTTGAAATATCACTGCTGGATGTTAAACTTGGGACAAAGA
CTTGCCGTAAAAAACCTTGGTTATCTTTGTGTATCTTTTATAGCTATAGTTTAAGCAA
TGAGACCTATGAGAAATCATTAGGACTTCTTAATGTACAAGATAATTTACACCGGTC
AGGTTTTGTTCTTGAGGGAGTTGTCCACGTTTATTTGTTTTGTATTACAGAAAACTT
TCTACCAGTTTCATAAAGCAATGAAATGTAAACTAGTAGCTCTTGGCTACAACCTT
GCTTTCCATATTTTGCTCATCTGACTTTCTGGTTCCAGTAATGTTTTGCCAGCTGCG
CGCAAGAAAGTGTCTTGCTTCTGTATTCATAGCTCTCAGTTTTCTGTATCATTTTCA
GCAATTTCAGGGCATTTCAAATTACTTGTTGCGATGTATATCCCAGAGATATGTGCT
TATTCTTTTAATTTATTTAAAGTAAAATGTAAATGAATTCGCTGCTGGTTTCATACAA
CAGAAATACATACTCCTTGTGTTGATGGTGTGTGTGTGTTTGTGTAATTAGAGTCCA
ATTTGTTTCTACGTGTGCATTGATGGTGTACCTGTCGTATTTGTTTTTGGAACCCCTT
ACAACTTCGAGTTTATATCCCTTAGGTGGTAGTACTATTAATTCCTTTCATTCCTTTC
ATTGTACTCCTGTTCATATTGTCTTTCTTCTGTCTTCCTTTCCACCCTTTCCTAACAA
TTGTTTCATATTGTAACTGTGAGGTTTTCCTCCTGTTACACTTTTGAAACCCTCTTTT

CTCTCAATTACCCATTCATCTCTGAATGACTTCATGGGTTCCAGCGCTTGGCCATTG
GCCTAAATTCTATATGTATTCCTTCCGTCAAGGTTATAGGTTATTTTATTATTACTAAT
TGTATTGATCTGATATTGGCATTTAATATGATGCAAGCACACTAACGCATCCAGTCA
CATGATTCTAGGCTTGCTTTATTTTCACCTTCCGATGACCATGTCACTGAGTGATGT
CTTATAAAATAGTCCTAAATGAATGAATTTTTCTATCCTGTGTCGATCGTGCAGAGT
TTCTCAGTTTGTATTATATATTTTTAACCACTTGCAATATTCATAAAATACTGTAGCAG
AAACATCGTTGGTGGTTCATAATTCATTTTGAATGAATGTTTATTATCACACTTCTGC
TTGCAAAGGTTATGCAACATTATCTAAACAGTAAAGACAGCAATGTATTGGTTTTTC
TGTGAAATAATTATTGCTGTAAAACTTTTAAAGGAATCATCTTATCATCTATATGAAT
TTTGCTGGTTTCGTATGCTATTTTATTCAAGCAAAAGACAACATTCTAGAAGTAAGG
CTGTGGAGAGAATTCTGTGAGTTAGATTTCATGTGGATGTTGTTGGTTATCTCTCTT
TCTGAGAAAGAAGCCAAAGACCGTACTGTAATGCATATATAAACTCAACTTGGTTT
AAGAAATGAAGGTATGTATTTGCAACTTTAAGTAGCGACACCACCCTACTTACAAA
TGAGTTACGTCCCAGATGGCTGTTTGTGTTGAATTTTTTTTGTACAGGTAGCCCTTG
GTTAACAGTGGGGCTTCCGCTGCTGTTAACTAGAAATTTTGCGCTGTTAACCGGAT
ATCGGCGCTGCTAACCAGAGATTAGCCCCAAAAATCCAGTTAACAGCGTCATTAGA
CAAGTGCTGTAAAACCGGATAGCCATTTAATGATACCGCCGTTAACCGAGAGCTGC
CTGTAGTTGGTTACTGTACTCTTCATGCCGATTTAAATACAGTATGATGTAAAATCA
GTACAGTTCTGTACGTTTTATCATCTTAGAACACAATCCACTGTACATACACTACTG
TATGTTTACAGTATGTATGGAGAGAATAGGCAAGCATAACAAAATTTGAAATTACG
GGTGGCAAAGGGGAGTGTTCGAAGACCACTCTAATTTACGATCACATTTGGTTGTG
TCTGAATGTTTGTAAGTATAGAATGTTCTTAAGTAGGGTGGTGTCTGTACTGTATTG
AGATATTTTTATGGCTCATAGTTCGAGGAGTTTTGCAAGTATTTTCTAATACTGTGTA
TTTCCGTTCTTTTAGGGTGTATAGTATGTTGCATGTGATTGTTATCATTATATTACTTT
ATTATTATTACTGTCATTATCATTGCTAAAAAAGTTACTGTGTCGTGACAGAACTGTT
CTTTGTTGAAAATCATCAGTCATTGCAATATCTTGGCTTGTAAGCTTTGACTGGAAT
ACAAGATACAATATGGGAACCAACTTTGGATTTTAGTGGCACCATCACAAACAAGA
GTACGTATTGTCCTACAATGCCAAGGGTCTCAGTAGTATATGGAAGATAAAAATTAA
GAATATACTATAAAAGAAAAATGAAAGATAGTAAGATGGCTGACTGTGAAATAAGA
CAGATGACATTATTCCAAACAAAAATAAACATTTGCACCAAGTTTTGAATTTCTGA
AGCCCCAAATTGTTGTGAAGCATAGACTCGTTTTTCCAGAATCTGTGCCTGCTGAC
TGCTGTGTTCATTAGTTTAGGTGTTTTTCCAGCCTAGTCATATTGAGAGTATGTTAGC
TTCTCTGCCGAGCGAGGCACCATTTTTGTAGAGATAATTAACCTCTTGTTCTTTCTG
AATTAGTATATATGATTTTGCAATTTTTTTTACTAATATATCAATGGCTGATCTGGGTG
TCACAGTTGTCGAGAAGTGAATATGAAATTGTGGTTACCAGTGGGATTCCTGTCTT
CTTTCCGTAGCCTATATTGTAGATTATAATCATAACTAGCTTTTTATATGTGGTACTGT
ACTCATAACGAATGAAATGATTCACACTGGTTTGAAAAGAAAATGCACTTGTTTGA
AAGAGCACTTCAGGGTTGAGTTTTGTGACAATGATATTGGCACAAAGAACATTTAG
AGTGAATGTATCACATTGCACCGACAAGTGAGACTCGGATAAAAGTTTATTGCAGT
ACAGTTTATTGTACGGTATGATTATAATTACTGTGTAATATTCAGTGAATAGGATTAG
GTTGTTTGTAAACAACTCCCGATTATGACACGTTAAAAATGAAGGACTCTTGTAAA
AAAAAAAAAAAA
